# Effectiveness of Fu’s subcutaneous needling combined with muscle energy technique for postpartum perineal pain: a randomized controlled trial

**DOI:** 10.3389/fmed.2025.1609500

**Published:** 2025-08-06

**Authors:** Yuchun Shao, Zhanxiang Lin, Yuan Shi, Ling He, Dongmiao Han, Zicai Liu

**Affiliations:** ^1^Department of Rehabilitation Medicine, Yuebei People’s Hospital, Shaoguan, Guangdong, China; ^2^Department of Rehabilitation Medicine, Shaoguan First People’s Hospital, Shaoguan, Guangdong, China; ^3^School of Rehabilitation, Gannan Medical University, Ganzhou, Jiangxi, China; ^4^Shaoguan First People’s Hospital, Shaoguan, Guangdong, China; ^5^Department of Rehabilitation Therapy Teaching and Research, Gannan Healthcare Vocational College, Ganzhou, Jiangxi, China

**Keywords:** Fu’s subcutaneous needling, muscle energy technique, postpartum perineal pain, randomized controlled trial, perineal rehabilitation, lateral episiotomy

## Abstract

**Background:**

Perineal pain after natural childbirth is a common problem among women after delivery, significantly impairing patients’ quality of life and increasing familial burden. This study evaluates the efficacy of Fu’s subcutaneous needling combined with muscle energy technique (FSN + MET) on perineal wound pain after natural childbirth.

**Methods:**

This randomized controlled trial was conducted at Yuebei People’s Hospital. Seventy women experiencing perineal wound pain following spontaneous vaginal delivery in the hospital’s Obstetrics Department between January 2023 and December 2024 were enrolled. Participants were randomly assigned to either an intervention group or a control group (35 per group) using a random number table. The control group received conventional rehabilitation plus MET, while the intervention group received FSN in addition to conventional rehabilitation and MET. Pain improvement was assessed after 5 days of treatment.

**Results:**

The efficacy of FSN + MET was assessed using the Number Rating Scale (NRS), Activities of Daily Living (ADL), Fugl-Meyer Lower Limb Motor Function Score (FMA), Self-Rating Anxiety Scale (SAS), and satisfaction survey of treatment effect. Before treatment, there was no significant difference between the two groups regarding age, days of gestation, postpartum pain scores, NRS score, FMA lower limb score, ADL, SAS, and satisfaction (*P* > 0.05). Following treatment, the FSN + MET group showed significantly greater improvements in NRS, FMA, ADL, and SAS scores compared to the control group (*P* < 0.05). This demonstrates that FSN + MET is superior to conventional methods in alleviating perineal wound pain, improving mobility, enhancing quality of life, and reducing anxiety in patients after spontaneous delivery.

**Conclusion:**

FSN + MET effectively reduces postpartum perineal pain and improves functional recovery.

## Graphical Abstract

Participant flow diagram: randomized trial of FSN + MET vs. MET in primiparous women. 
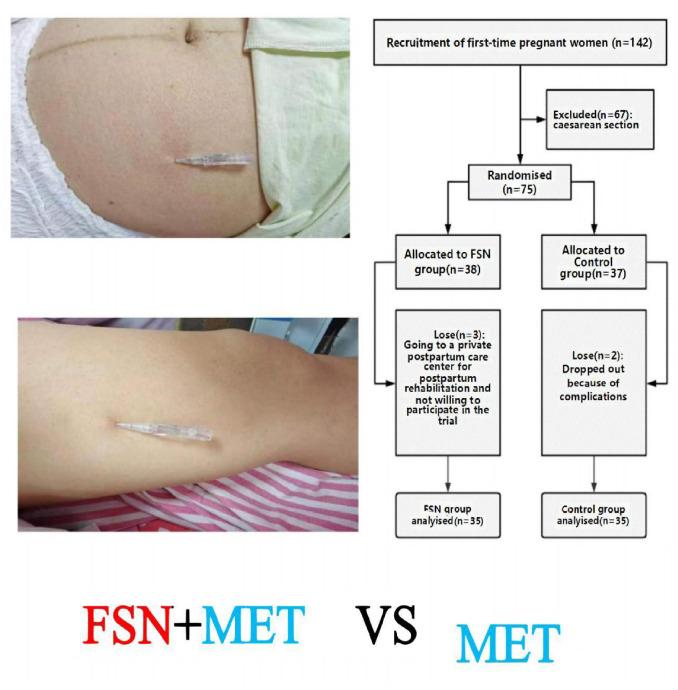

## 1 Introduction

During spontaneous maternal labor, medical personnel usually perform episiotomy to avoid crushing the baby’s head and maternal birth canal tear injury during labor (1, 2). Episiotomy is routinely performed by obstetricians and midwives and is based on the principle of preventing extensive perineal tears and increasing the diameter of the vaginal outlet to facilitate the birth of the baby (3). Some studies have shown that the rate of perineal lateral incision in women who deliver vaginally can be as high as 40% (4). Lateral episiotomy can lead to a series of complications such as hemorrhage, fever, pain, wound infection, swelling, wound dehiscence, and secondary pelvic floor dysfunction, which can seriously impair maternal physical and mental health. Perineal pain is the most common complication in postpartum women undergoing episiotomy (5). Perineal pain not only affects a woman’s physical recovery, such as discomfort with urination, defecation, and sexual intercourse (6), but also affects early neonatal care. Perineal pain may last for days or even months (7). Many factors may influence the severity of perineal pain, such as the number of births, the type of labor, the birth experience, the degree of perineal trauma, and the birth weight of the newborn, and studies have shown that mothers who have had a lateral incision are also twice as likely to experience pain as those who have not had the procedure (8).

Conventional clinical treatments include chiropractic (9), physiotherapy, and medication (REF) (10). Most women take non-steroidal anti-inflammatory drugs for pain control after delivery, however, oral medications may have side effects such as ulcer formation, nausea, vomiting, and renal insufficiency (11). In addition, prescribing pain medication during breastfeeding is controversial and may lead to adverse drug reactions in the newborn and infant (12). Studies have shown that physiotherapy such as infrared light and laser have no benefit (13); perineal cold compresses can be effective in relieving pain, however, patients cannot apply cold compresses for a long period; topical medications need to be routinely sterilized, and the site of the perineal area is hidden, which will increase the burden of care, and is not easy to be accepted clinically due to the restriction of the mother’s movement after delivery. Currently, there is no standardized clinical intervention for postpartum perineal pain, highlighting the need for innovative and effective treatment strategies. Fu’s subcutaneous needling (FSN) is a modern acupuncture-derived therapy (14) that involves inserting a specialized needle subcutaneously to stimulate superficial fascial tissues mechanically. Unlike traditional acupuncture, FSN focuses on loosening tight fascial layers and “affected muscles” (muscles contributing to pain distal to the injury site) through sweeping motions of the needle. This technique aims to modulate bioelectrical activity, improve local blood flow, and alleviate pain without causing discomfort during treatment (14, 15). Muscle energy technique (MET) is a manual therapy method where patients actively contract targeted muscles against controlled resistance applied by the therapist. This process facilitates muscle relaxation, enhances joint mobility, and reduces pain through reciprocal inhibition and mechanoreceptor activation. It has established efficacy in postpartum rehabilitation. MET improves musculoskeletal function by engaging patients in controlled isometric contractions against therapist-applied resistance, which enhances proprioception, reduces muscle stiffness, and promotes pain relief. Prior studies have demonstrated its effectiveness in alleviating postpartum pelvic pain and improving functional mobility (16).

At present, few studies have combined the two techniques to treat postpartum vulvar wound pain. So, what is the efficacy of FSN + MET for postpartum vulvar pain? This study uniquely combines these techniques, aiming to evaluate their synergistic effects on perineal pain management.

## 2 Materials and methods

### 2.1 Study design

#### 2.1.1 General information

Patients with painful perineal wounds were recruited from January 2023 to July 2023 who delivered their babies in the Obstetrics Department of Yuebei People’s Hospital, and the cases that met the inclusion criteria were randomized and assigned using the random number table method. Outcome assessors and statisticians were blinded to group allocation. Treatment providers were unblinded due to the nature of FSN. This study was supported by the Guangdong Provincial Bureau of Traditional Chinese Medicine Scientific Research Project (20242088) for the year 2024, Guangdong Shaoguan City Health and Health Research Projects (Y24098), and the Shaoguan Municipal Health and Wellness Scientific Research Project (Y23044) for the year 2023, which was recorded in the National Health Protection Information Platform/Medical Research Registration and Filing Information System. The research has been filed in the National Health Protection Information Platform/Medical Research Registration and Filing Information System (No. MR-44-23-045676).^1^ All processes comply with CONSORT guidelines and follow the Declaration of Helsinki.

#### 2.1.2 Diagnostic criteria

The criteria for women in labor were developed based on the Royal College of Obstetricians and Gynecologists RCOG (2015) guidelines (17).

#### 2.1.3 Inclusion criteria

1. Adults, female, aged 21–40 years;
2. Patients with perineal tear or lateral incision delivered by normal delivery;
3. The patients are conscious, without mental illness, and can well cooperate with the experimenter to complete the experimental operation;
4. Subjects were informed in advance of the experiment-related matters and signed an informed consent form.

#### 2.1.4 Exclusion criteria

1. Patients with combined primary diseases of cardiovascular, cerebrovascular, hepatic, renal, and hematopoietic systems;
2. Combined with prenatal infection and acute vaginitis;
3. Patients with infection at the site of needling;
4. Presence of conscious cognitive impairment or serious mental illness;
5. Those who are unwilling to sign the informed consent and refuse to participate in this study.

#### 2.1.5 Efficacy evaluation criteria

The main observation indices were the Number Rating Scale (NRS), Activities of Daily Living (ADL), Fugl-Meyer Lower Limb Motor Function Score (FMA), Self-Rating Anxiety Scale (SAS), and satisfaction survey of treatment effect.

**NRS:** 0 means no pain; 1–3 means mild pain; 4–6 means moderate pain; 7–10 means severe pain, and the higher the number, the more severe the pain.

**ADL:** <20 points: total disability; 40–21 points: heavy dependence; 60–41 points: moderate dependence; >60 points: mild dependence, basic self-care; and 100 points can be a completely self-care life.

**FMA:** Items related to lower extremity functions in daily life, such as bathing, walking, putting on shoes, socks, etc.; 20 items are included, with each item having a score of 0–4 and a maximum score of 80; the lower the score, the more significant the disability.

**SAS:** 50–59 indicates mild anxiety, 60–69 indicates moderate anxiety, and 70 or more indicates severe anxiety. The higher the score, the more obvious the anxiety.

Treatment effect satisfaction survey: Patients rated this treatment service as very satisfied, satisfied, generally satisfied, and unsatisfied.

#### 2.1.6 Statistical methods

The analysis included measures such as mean (standard deviation) for normally distributed variables, median (IQR) for non-normally distributed variables for continuous variables, and frequencies and percentages for categorical variables. Normality tests and QQ plots were used to assess the data distribution, and appropriate descriptive statistics methods were applied to both normally and non-normally distributed variables. Group comparisons for continuous variables with normal distribution were performed using Welch’s *t*-test or ANOVA. Non-normally distributed variables were compared using the Wilcoxon rank-sum test or the Kruskal–Wallis test. For comparison between groups of categorical data, we used the Fisher’s exact test for expected frequencies < 5; otherwise, we used the Chi-squared test.

In our study, all statistical analyses were performed using the R software (version 4.2.2). The sample size of subjects required for this study was calculated by the statistical software G*Power 3.1.9. Effect size (*d* = 1.05) was derived from prior FSN studies (18) (VAS reduction: 1.50 ± 1.48 vs. 2.97 ± 1.30). With α = 0.05 and power = 97%, 56 subjects were required. Accounting for 20% attrition, 70 were enrolled.

### 2.2 Treatment programs

#### 2.2.1 Control group

All patients in the control group were treated routinely, firstly, the perineum was routinely sterilized with iodine povidone, and then a disposable medical ice bag (QinXin, 12 cm × 36 cm) was stuck in the underwear or other clean fabrics as a cold compress on the perineum/wound; after the cold sensation disappeared, the ice bag was disposed of in the bucket of dirt for 3 times/day, and the perineal wound was irradiated with the Specific Electromagnetic Wave Therapeutic Apparatus (Chongqing Shuxuan Medical Equipment Co., Ltd., CQX-23D, a.c.220V 50 Hz, 250 W) (19), parameters included a wavelength of 2–25 μm and intensity of 35 mW/cm^2^; 24 h later to promote wound healing and blood circulation, and reduce pain. Two times per day of treatment; during the period of treatment, the medical staff always pays attention to the affected area of the patient to avoid frostbite or burn. The equipment selection was based on hospital protocols, and potential variations in device efficacy were mitigated by using identical models across all participants.

MET [15]: MET as an intervention was initiated after enrollment of the trial patients. Improvement of musculoskeletal system function and pain reduction is achieved through patient-initiated participation utilizing isometric muscle contraction against resistance. The level of resistance during MET was equivalent to grade 4 of this patient’s unarmed muscle strength, and both MET and floating needle maneuvers were performed by the same therapist. Therapists applied force equivalent to 20%–30% of the patient’s maximal voluntary contraction, as assessed during baseline evaluations. All therapists underwent standardized training to maintain inter-operator consistency, including pre-trial workshops on force calibration and patient positioning. During the treatment, the therapist would precisely control the direction and the amount of force applied. Patients were instructed to do ankle dorsiflexion and plantarflexion, knee flexion and straightening, hip flexion and straightening, hip adduction and abduction under 45° of hip flexion, abdominal breathing, etc., and to perform appropriate resistance exercises with the operator. This method improved the symptoms of walking-induced perineal pain in the patients for 1 time-d-1, each time for 15 min, and was done for 5 days in a row, and patients in the control group received the MET for a total duration of 75 min.

#### 2.2.2 Experimental group

On the basis of the operation of the conventional control group, the floating needle was applied for intervention. First of all, the determination of the affected muscle was carried out, concerning the “Compendium of Floating Needle Medicine,” the patients were palpated along the medial patella to the root of the medial thigh, and along the umbilicus level of the abdomen to the medial anterior superior iliac spine, and there was a tightness, stiffness, hard slippery sensation, cable, hard joint sensation under the finger, and the localized acidity and discomfort could be determined as the affected muscle (20), mainly the iliopsoas muscle, rectus femoris, adductor magnus muscle, abdominis muscle, and pubic muscle.

#### 2.2.2.1 Operation method

**FIGURE 1:**
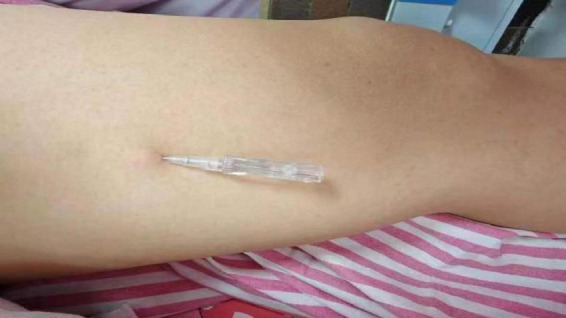
Fu’s subcutaneous needling treatment on inner thighs.

**FIGURE 2:**
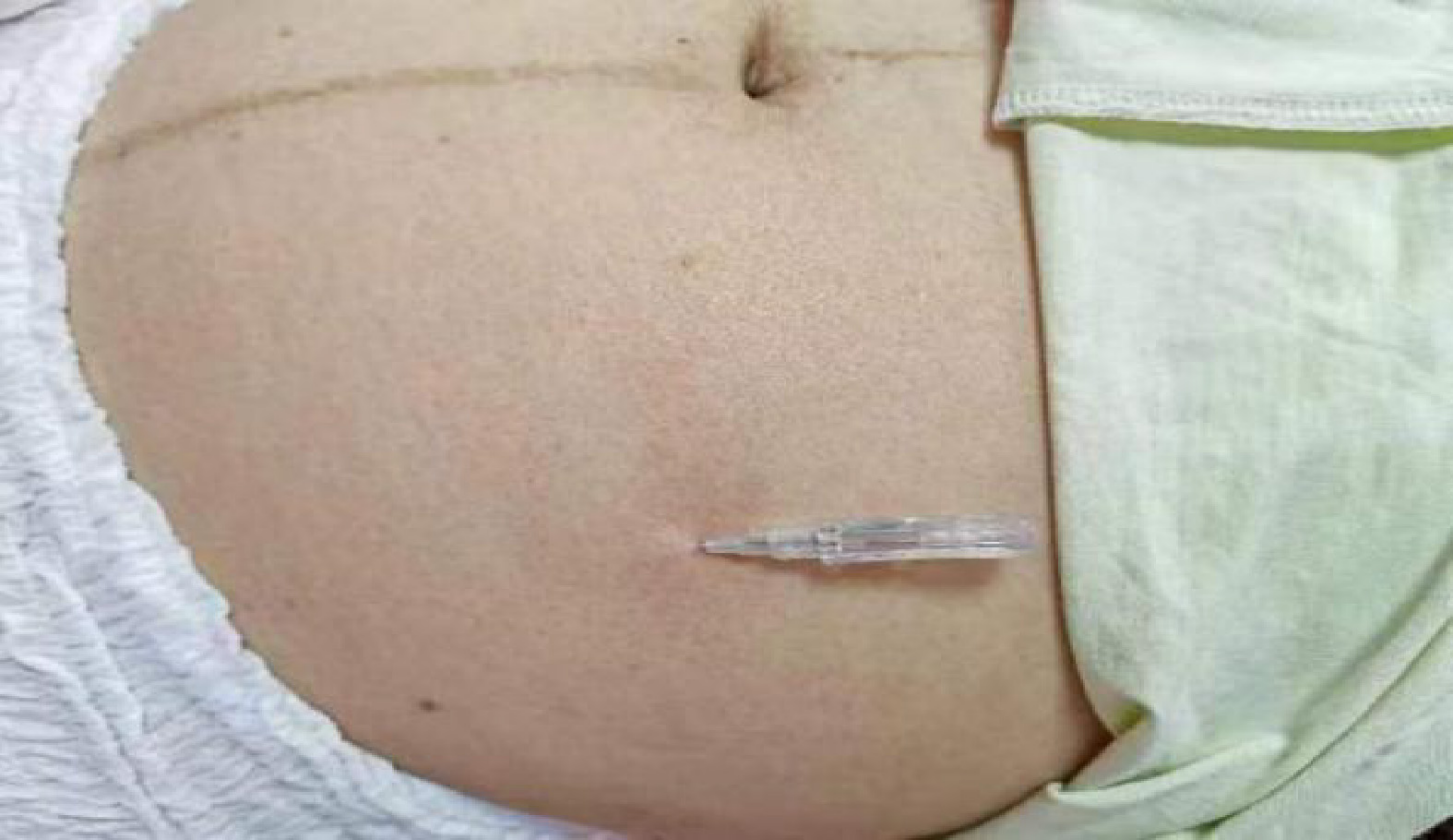
Fu’s subcutaneous needling treatment in the abdomen.

The M-size single-use floating needle (Huaguan brand) was used. Operation (21): (1) patients take a prone position, popliteal fossa under the pillow so that the patient’s lower limbs are in a relaxed state, in the inner thigh on the medial edge of the patella 15 cm, to the inner thigh in the direction of the perineum, to determine the point of entry of the needle, as shown in Figure 1, the routine disinfection of its with the help of the floating needle into the subcutaneous to ensure that the tip of the needle is always in the subcutaneous after the right hand to hold the needle along the subcutaneous forward to keep the needle body is slightly lifting the needle tip cocked exit 0.4 cm needle core handheld needle seat. (2) In the patient’s abdominal anterior superior iliac spine of anterior superior iliac spine. Slightly cocked exit 0.4 cm needle core holding the needle holder. (3) In the patient’s abdomen 10 cm medial to the anterior superior iliac spine or lower abdomen near the midline 15 cm, to determine the needle point, as shown in Figure 2, the method of injection is as above. (4) Sweeping and dispersing the needles in a fan shape for the two points of needle insertion, with a sweeping frequency of about 200 times/min, and a sweeping time of about 3 min for each point of needle insertion. When sweeping and dispersing, the movement was gentle, and the patient should have no sensation of acidity, numbness, distension, pain, and so on. MET (22) is combined with sweeping to enhance the therapeutic effect, and patients are instructed to do ankle dorsiflexion, plantarflexion, knee flexion, extension, hip flexion, extension, hip flexion, and hip adduction at 45°, abdominal breathing, and appropriate resistance exercise with the operator so that the patient’s muscles are in a contracted and tense state for 10–15 s. The operator applies a soft force, which should not be too large, and continuously observes the patient’s situation and adjusts the operation time. Keep observing the patient’s condition and adjust the operation time. For each muscle, 2–3 times of resistance exercise should be carried out to assess the patient’s treatment condition and treatment effect. After 15 min, the above operation should be carried out again for the patient, and the patient’s symptoms will be relieved, which means that the treatment is successful. (5) Assess again whether the patient’s symptoms have improved, and touch the affected muscle to see whether the tightness, stiffness, hardness, slippery feeling, hard knots, and stripes have been relieved or disappeared. (6) Withdraw the needle core, leave the plastic hose under the skin, seal the mouth of the hose with a medical bandage, ask the patient not to get wet, no strenuous exercise, carry out normal daily activities with the hose, and leave it for 4∼6 h before pulling out the hose, 1 time/day, floating needle was performed on the first and third days of the 5 days, with a total of 2 float treatments, each with a total of about 30 min of sweeping and 4–6 h of needle retention in conjunction with MET exercises.

## 3 Results

### 3.1 General information

**FIGURE 3:**
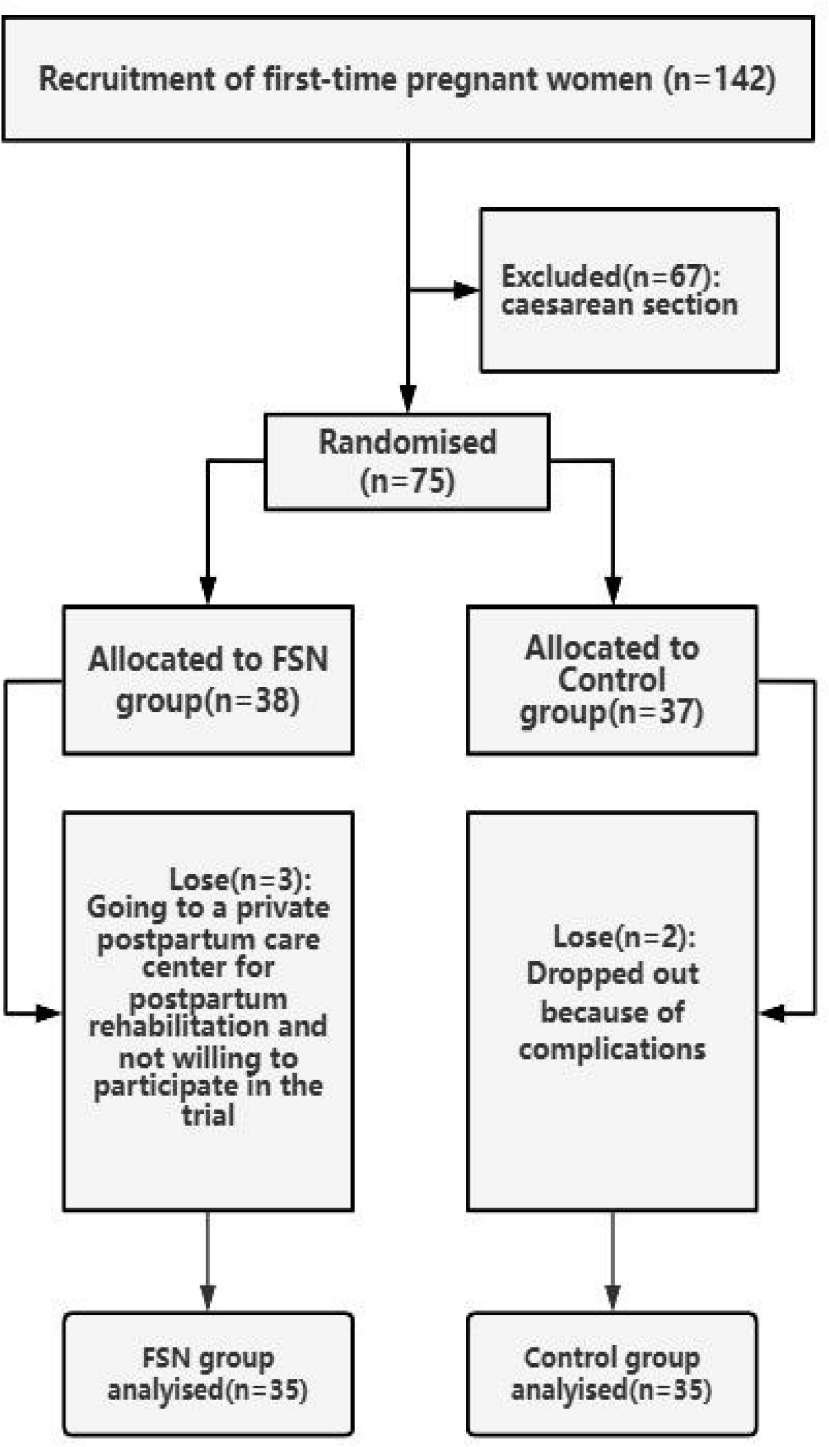
Trial flowchart for enrolled patients.

During the study period, there were 142 primiparous women. Of these, 75 were delivered vaginally, and 67 subjects underwent cesarean section. Seventy-five individuals with postpartum perineal pain who met the inclusion criteria were randomly assigned to the FSN group and the control group. Five subjects withdrew from the study due to personal reasons (e.g., relocation and inability to attend follow-up sessions). These dropouts were unrelated to the treatment protocols. Finally, 70 women were enrolled in the trial. Through the randomized numerical table method, 35 women were assigned to the control group to receive conventional treatment only, and another 35 women were included in the FSN group for flotation treatment under conventional care and the flow chart of the included patients is shown in Figure 3.

The minimum age of the included patients was 22 years, and the maximum age was 37 years; the minimum duration of postpartum pain was 2 days, and the maximum duration was 10 days. Detailed baseline information is shown in Table 1. The baseline characteristics of participants in both the FSN and Control groups were generally similar across the measured parameters, with no statistically significant differences observed. The mean age was slightly higher in the FSN group (29.0 ± 3.4 years) compared to the Control group (29.3 ± 3.4 years), although this difference was not significant (*P* = 0.649). The median days of pregnancy were also comparable between the FSN group (279 days) and the Control group (278 days), with a *P*-value of 0.865. The mean value of the number of days of postpartum pain in the two groups was 4.03 ± 1.65 days vs. 4.54 ± 1.88 days, indicating that the stage of the pain course at the time of the first intervention was comparable in both groups (*P* = 0.184). Furthermore, NRS, Fugl-Meyer, and ADL scores were nearly identical across the groups (all *P* values > 0.8), indicating the uniform distribution of these characteristics at baseline. For the categorical variables, the percentage of participants with the characteristic “satisfied” was higher in the FSN group (20.0%) compared to the Control group (11.4%), while the characteristic “Generally satisfied” had an equal distribution of 48.6% in both groups, and the characteristic “Unsatisfied” was more prevalent in the Control group (40.0%) than in the FSN group (31.4%). Despite these variations, the lack of statistically significant findings suggests a balanced baseline between the FSN and Control groups, allowing for a fair comparison of subsequent outcomes. No significant differences in SAS scores were found (*P* = 0.711), further supporting the equality of the groups at baseline.

**TABLE 1:** Patient demographics and baseline characteristics.

Characteristic	Group		*P*-value
	FSN group, *N* = 35^1^	Control group, N = 35^1^	
Age	29.0 ± 3.4	29.3 ± 3.4	0.649^2^
Days of pregnancy	279.0 (272.0, 283.0)	278.0 (274.0, 283.0)	0.865^3^
Postpartum/day	4.03 ± 1.65	4.54 ± 1.88	0.229^2^
NRS	7.00 (6.00, 8.00)	7.00 (6.00, 8.00)	0.867^3^
Fugl-Meyer	21.03 ± 3.05	21.17 ± 3.24	0.850^2^
ADL	30 (20, 40)	30 (15, 45)	0.920^3^
Satisfaction			0.555^4^
Satisfied	7 (20.0%)	4 (11.4%)	
Generally satisfied	17 (48.6%)	17 (48.6%)	
Unsatisfied	11 (31.4%)	14 (40.0%)	
SAS	57 (47, 67)	57 (51, 69)	0.711^3^

NRS, Number Rating Scale; ADL, Activities of Daily Living; FMA, Fugl-Meyer Lower Limb Motor Function Score; SAS, Self-Rating Anxiety Scale. ^1^Mean ± SD; median (IQR); *n* (%). ^2^Welch two sample *t*-test. ^3^Wilcoxon rank-sum test. ^4^Pearson’s Chi-squared test.

### 3.2 Comparison of NRS/FMA/ADL/SAS scores between the two groups of patients following treatment

Before the intervention, there was no significant difference between the two groups’ NRS/FMA/ADL/SAS scores (*P* > 0.05, see Table 1), and we used the changes in outcomes before and after the treatment to compare the differences between the two groups. The results showed that the NRS/FMA/ADL/SAS scores of the patients in the observation group were significantly different from those of the control group (*P* < 0.05, see Table 2). It showed that FSN + MET improved more than traditional rehabilitation therapy.

**TABLE 2:** Difference in change from baseline after treatment between the two groups.

Change from baseline	Group		*P*-value
	FSN group, *N* = 35^1^	Control group, *N* = 35^1^	
NRS	5.00 (4.00, 5.00)	2.00 (2.00, 3.00)	<0.001^2^
FMA	10.0 (8.0, 12.0)	6.0 (4.0, 8.0)	<0.001^2^
ADL	50 (45, 65)	30 (20, 35)	<0.001^2^
SAS	33 (23, 39)	20 (12, 26)	<0.001^2^

NRS, Number Rating Scale; ADL, Activities of Daily Living; FMA, Fugl-Meyer Lower Limb Motor Function Score; SAS, Self-Rating Anxiety Scale. ^1^Median (IQR). ^2^Wilcoxon rank-sum test.

### 3.3 Comparison of satisfaction evaluation between the two groups before and after treatment

Before the intervention, there was no significant difference in the satisfaction evaluation of the two groups (*P* > 0.05), and after the treatment, there was a significant difference in the satisfaction evaluation of the observation group compared with the control group (*P* < 0.05), indicating that FSN + MET showed greater improvement in patient satisfaction relative to conventional treatment (Table 3).

**TABLE 3:** Comparison of satisfaction evaluation before and after treatment between the two groups of patients.

Characteristic	Group		*P*-value
	Control group, *N* = 35^1^	FSN group, *N* = 35^1^	
**Baseline satisfaction**			
Very satisfied	0	0	0.555^2^
Satisfied	4 (11.4%)	7 (20.0%)	
Generally satisfied	17 (48.6%)	17 (48.6%)	
Unsatisfied	14 (40.0%)	11 (31.4%)	
**Post-treatment satisfaction**			
Very satisfied	7 (20.0%)	26 (74.3%)	<0.001^3^
Satisfied	10 (28.6%)	8 (22.9%)	
Generally satisfied	17 (48.6%)	1 (2.9%)	
Unsatisfied	1 (2.9%)	0 (0.0%)	

^1^*n* (%). ^2^Pearson’s Chi-squared test. ^3^Fisher’s exact test.

### 3.4 Typical case

The patient is female and 27 years old, with pregnancy 1 and delivery 1. Admission: 4 July 2023; Complaint: menopause 38 weeks + 3 days, bloody vaginal discharge for 2 hours. History: on 5 July 2023, she gave birth to a live baby and was given a perineal lateral cut and suture. She was bedridden and had difficulty bending her hips and knees and lifting her buttocks, and was unable to transfer from the prone to the sitting position; her pain was scored at 8 on the NRS, and she was given ice packs, but there was no significant improvement. On the second day after delivery, she was referred to the Department of Rehabilitation Medicine for a consultation. Consultation opinion: the patient had pain in the perineal lateral incision wound after delivering a normal delivery, and ice packs were ineffective, so FSN treatment was given to relieve the pain. Before treatment, the patient was examined and evaluated, and the affected muscles were searched and palpated in the lower part of the rectus abdominis muscle (++++), internal and external abdominal obliques (++), iliopsoas muscles bilaterally (++++), and the middle and upper part of the internal femoral group bilaterally (++++). Treatment: using a disposable FSN, the above-affected muscles were treated sequentially. The needle entry point of the abdomen was chosen to be about 10 cm from the medial margin of the iliac spine and about 15 cm from the medial margin of the patella of the inner thigh, and all the needles were pointed to the perineum, and sweeping and dispersing motions were carried out for 2–3 min, combined with the MET, and the condition of the patient was constantly monitored during the treatment, to prevent the occurrence of emergencies. At the end of the operation, the patient reported that the pain was reduced and the effect was satisfactory, and the patient was evaluated again, the lower part of bilateral rectus abdominis muscle (+), internal and external abdominal obliquity muscle (+), bilateral iliopsoas muscle (++), the upper part of bilateral femoral internal retractor group (++), and the affected muscles’ pain score of VAS score of 4, the core of the needle was taken out, and the hose was left subcutaneously, and the hose was fixed by medical adhesive tape for 6–8 h, and then the medical personnel was asked to remove it. On 7 July 2023, the second consultation: the patient’s pain improved significantly, the effect was very satisfactory, the pain score of the affected muscle VAS score 1, palpation of the affected muscle bilateral rectus abdominis lower part, internal and external abdominal obliques, bilateral iliopsoas (+), bilateral femoral adductors upper part (+), the affected muscle tension, rigidity improved significantly. On 8 July 2023, the patient was discharged from the hospital, complaining of no obvious pain. The patient was followed up for half a month without recurrence.

## 4 Discussion

Postpartum women undergoing episiotomy often experience perineal pain that lasts for months during the first few hours after birth. This study demonstrates that FSN + MET effectively reduces perineal pain following natural childbirth and lateral incisions. FSN + MET improves the ability to perform daily living activities, improves lower limb walking function, and relieves maternal psychological anxiety in patients with postpartum perineal pain, relative to conventional treatment. It is reported that this is the first clinical randomized controlled trial using FSN + MET to treat postpartum labor and delivery vulvar wound pain, and it is hoped that it can provide a reference for clinical postpartum rehabilitation treatment.

Natural delivery is strongly recommended by modern obstetrics and gynecology (23) due to its advantages for both the mother and the newborn. It promotes a quicker recovery for the mother and enhances the fetus’s ability to adapt to the external environment after birth. However, with the improvement of living standards, many pregnant women lack awareness of the importance of health management concerning body weight. This can result in various complications during pregnancy, such as diabetes, obesity, and excessive fetal size, all of which can negatively impact the birth process. Lateral episiotomy is a good solution to these problems and shortens the second stage of labor. However, there are a series of other postoperative problems, such as the difficulty of “second bowel movement,” wound infection, edema, pain, psychological anxiety, irritability, reduced breastfeeding and milk secretion, pelvic floor dysfunction, and prolonged hospitalization (24). Relevant studies (25) showed that edema and pain of lateral perineal incision may lead to poor healing and infection of the perineal incision, which has a great impact on maternal recovery, so it is important to pay attention to the effective treatment of edema and pain of the perineal wound.

Fu’s subcutaneous needling differs from traditional acupuncture in both theory and application. While traditional acupuncture targets meridians and specific acupoints to regulate Qi, FSN focuses on loosening superficial fascia and “affected muscles” distal to the pain site. FSN uses unidirectional needle sweeping in subcutaneous layers without eliciting De Qi sensations, reducing patient discomfort. This approach may offer faster pain relief for postpartum perineal pain compared to traditional methods, which often require deeper needle insertion and longer retention times. However, FSN’s reliance on fascial manipulation may limit its applicability in cases of deep tissue pathology, where traditional acupuncture’s holistic effects could be advantageous. The pelvic floor muscles are an important part of the core stabilizing muscles that form the base of the abdominal cavity. These muscles must contract in different tasks to increase intra-abdominal pressure to maintain self-control and lumbopelvic stability (26). MET is the process of precisely controlling the direction and varying degrees of voluntary contraction of the patient’s muscles so that the patient counteracts the therapist’s apparent counterforce, avoiding pain by interactively inhibiting and stimulating mechanoreceptors (27).

Modern fasciology says that (28) pain is closely related to muscle and fascial tissue, and when a painful lesion occurs in a certain muscle, its attached fascia will then have functional structural changes. Therefore, FSN therapy (29) puts forward the theory of “affected muscle,” which explains that the muscle at the focal site may not necessarily be the affected muscle but may be the peripheral or distal muscle that causes its pain, and FSN therapy mainly stimulates the subcutaneous superficial fascia around the limited pain (30) to treat the affected muscle. The theory of “affected muscle” was proposed by FSN therapy (29), which stated that the muscle at the focal site is not necessarily the affected muscle, but may be the surrounding or distal muscles that cause the pain. In this study, from the theory of FSN medicine, the pain of patients with perineal wound pain appeared in the perineum, but the treatment sites were in the rectus abdominis and internal and external abdominal obliques on the medial side of the anterior superior iliac spine in the lower abdomen and the intramuscular femoral muscles on the medial side of the thighs in the lower limbs. The affected muscles in these areas are selected through treatment to loosen the fascial layer to achieve rapid pain relief, thus improving the patient’s ability to take care of himself. From the perspective of modern medicine, the effect of FSN therapy in treating the loosening of the affected muscles (31), maybe in the vicinity or distal end of the affected muscles into the needle sweeping stimulation to the subcutaneous loose connective tissues, the local generation of bioelectricity conduction to the lesion, improve the cellular ion channels (32), and combined with the MET, through several muscle contraction and relaxation activities, so that the lesion site of the blood circulation has been significantly improved to accelerate the metabolism of the material and thus improve muscle function and relieve pain symptoms. Current research on floating needles has yielded benefits in pain and a wide range of disorders (33–36). FSN may modulate pain via mechanical stimulation of superficial fascia (37–39), altering bioelectrical activity (40), and local blood flow (41). Synergy with MET’s neuromuscular inhibition could disrupt peripheral sensitization and reduce central pain signaling (42–44). Compared to cold therapy or infrared light, FSN + MET offers sustained relief without temperature-dependent limitations. Unlike pharmacotherapy, it avoids systemic side effects, making it suitable for breastfeeding mothers.

This study has the following limitations: no rigorous sample size calculations were performed, and the sample size may be insufficient, but existing studies have shown a trend of positive effects. In addition, our evaluation indexes lacked electrophysiological testing indexes such as electromyography, and the outcome indexes used were manually measured scales, which may lack objectivity and have a certain subjective bias. Subsequent studies should incorporate objective electrophysiological measures (e.g., electromyography) to validate subjective pain and function scales. Multicenter trials with diverse populations and longer follow-up periods are needed to assess the long-term efficacy of FSN + MET. Additionally, mechanistic studies exploring fascial remodeling and neuromuscular adaptations post-FSN + MET could elucidate the biological basis of observed improvements.

## 5 Conclusion

The results of this study show that FSN + MET can effectively improve the pain of patients after normal labor and delivery, and the improvement of quality of life, lower limb walking function, and anxiety is significantly better than the conventional treatment, which provides a reference basis for clinical postnatal rehabilitation treatment. This method is easy to operate, safe, and reliable, and deserves further promotion in clinical practice.

## Data Availability

The original contributions presented in this study are included in this article/supplementary material, further inquiries can be directed to the corresponding authors.

## Footnotes

1
https://www.medicalresearch.org.cn
